# The Utilization of Spironolactone in Heart Failure Patients at a Tertiary Hospital in Saudi Arabia

**DOI:** 10.7759/cureus.10032

**Published:** 2020-08-25

**Authors:** Abdulmalik S Alotaibi, Numan Alabdan, Abdullah M Alotaibi, Haifa Aljaafary, Mohammed Alqahtani

**Affiliations:** 1 Pharmaceutical Care, King Abdullah International Medical Research Center/King Saud bin Abdulaziz University for Health Sciences/King Abdulaziz Medical City – Ministry of National Guard Health Affairs, Riyadh, SAU; 2 Medicine, King Abdullah International Medical Research Center/King Saud bin Abdulaziz University for Health Sciences/King Abdulaziz Medical City – Ministry of National Guard Health Affairs, Riyadh, SAU

**Keywords:** heart failure, spironolactone utilization, aldosterone antagonists, mineralocorticoid receptor antagonist, saudi arabia

## Abstract

Introduction: Heart failure (HF) has high morbidity and mortality rates. Spironolactone has shown a 30% reduction in all-cause mortality, reduction in hospitalizations, and sudden death. However, data shows low use of spironolactone in HF patients. We aim to assess spironolactone utilization in HF reduced Ejection Fraction (HFrEF) patients and to identify the factors affecting its prescribing.

Methods: A retrospective cross-sectional study of patients diagnosed with HF from January 2016 to January 2017 conducted at King Abdulaziz Medical City-Riyadh. Inclusion criteria: all adult HFrEF <40% who are eligible for spironolactone with New York Heart Association (NYHA) class II-IV. Serum creatinine should be <2.5 mg/dL in men or <2.0 mg/dL in women, or estimated glomerular filtration rate (eGFR) >30 mL/min/1.73m^2^ and potassium <5.0 mEq/L. Exclusion criteria: pediatrics, end-stage renal disease, primary aldosteronism, and allergy to spironolactone.

Results: We screened around 5000 HF patients, of whom 368 were included. Among 195 patients who were not on spironolactone, 121 patients were eligible to use it; however, they did not receive it. One hundred seventy-three patients were on spironolactone, of whom 30 received the drug although they did not meet the eligibility criteria. The mean age of patients on spironolactone was 61±14 and the mean age of patients not on spironolactone was 66.6±15.6. Two hundred seventy-seven patients in the study population were male. Regarding comorbidities, 265 patients were diabetic. As for laboratory findings, the mean potassium for patients on spironolactone was 4.3 mEq/L; the creatinine and eGFR for patients on spironolactone were 82 umol/L (0.9 mg/dl) and 88 mL/min/1.73m^2 ^while those not on spironolactone had higher creatinine at 93 umol/L (1 mg/dl) and eGFR 80 mL/min/1.73m^2^. Using multivariate regression, we found many factors affecting spironolactone utilization, including EF before spironolactone, serum creatinine, angiotensin-converting enzyme inhibitors (ACEI), angiotensin-II receptor antagonists (ARBs), furosemide, statin, and stroke.

Conclusions: Spironolactone for HFrEF is underutilized. EF before spironolactone, serum creatinine, ACEI, ARBs, furosemide, statin, and stroke significantly affect spironolactone utilization. Further studies are warranted to identify barriers affecting spironolactone utilization in HF patients from prescribers' perspectives.

## Introduction

Heart failure (HF) is a common clinical condition with growing incidence and prevalence manifested by fatigue, dyspnea, and volume overload [1]. Worldwide, HF affects around 26 million people. In the United States (US) around 5.8 million people have HF. In addition, approximately 20% of the US population will develop HF in their lifetime [2]. Moreover, because of its increasing incidence, in 2030, HF will be diagnosed in one in every 33 US citizens, which could be linked to the aging population and the rate of survival for patients after myocardial infarction with current treatment options [3,4]. In Saudi Arabia, the estimated prevalence of HF is about 455,222 cases with an annual incidence of 32,200 cases [5]. Additionally, HF poses a tremendous burden with approximately 1.04 billion USD for a total of 320,000 HF-treated patients (8,137 USD annual cost per patient) [6]. Besides its high mortality and significant morbidity, HF patients will require multiple hospitalizations. It is responsible for nearly 1 million annual hospitalizations in the US and more than 12 million physician office visits every year [7,8]. Efforts have been done to optimally implement cost-effective and high-value therapies to improve overall provided care and reduce cost expenditures [9].

Currently, many therapeutic options exist for treating HF, including angiotensin-converting enzyme inhibitors (ACEIs), angiotensin-II receptor antagonists (ARBs), aldosterone antagonists (AA), ß-blockers (BB), and angiotensin receptor-neprilysin inhibitor (ARNI). Many trials have shown the added benefits of AA, commonly spironolactone, which has shown a 30% reduction in all-cause mortality, in addition to a reduction in hospitalizations and sudden death [10,11]. Based on that, it has a high-class recommendation by the American College of Cardiology/American Heart Association (ACCF/AHA) and the European Society of Cardiology (ESC) guidelines for chronic HF [12,13]. To promote the safe practice, guidelines have specified that before starting patients on spironolactone, serum creatinine levels should be 2.5 mg/dL or less and 2.0 mg/dL or less in men and women, respectively. Also, serum potassium levels should be less than 5.0 mEq/L. However, gaps in utilizing and monitoring spironolactone have been identified and addressed in many studies [14-18]. In a large 2009 observational study of more than 40,000 patients, less than one-third of eligible HF patients received AA [18]. Another study with a sample of 15,381 patients found that only 36% had a prescription of AA at discharge [19]. It has been suggested that the primary reasons for failure to prescribe AA are physicians’ knowledge, familiarity, and agreement with guidelines [18]. In support of this hypothesis, another study found that 9% of primary care providers (PCPs) were not familiar with eplerenone [20]. Besides, the safety monitoring for hyperkalemia has been found inadequate as shown in the Randomized Aldactone Evaluation Study (RALES); since there was a noticeable increase in hospital admissions for hyperkalemia and in-hospital deaths [21]. One reason that may contribute to this finding is the lack of close monitoring of potassium levels in patients receiving spironolactone. Moreover, Shah et al. concluded that patients on spironolactone for HF do not receive needed follow-up of potassium or creatinine concentrations, taking into consideration that hyperkalemia and renal dysfunction are common [17].

These findings shed light on the aims of this study, which are to assess the utilization of spironolactone in indicated HF patients, to provide insights about the current practice in Saudi Arabia, and to identify factors limiting the use of AA.

## Materials and methods

This was a retrospective cross-sectional chart review of patients with HF diagnosis from January 2016 to January 2017 conducted at King Abdul-Aziz Medical City (KAMC) in Riyadh. Inclusion criteria involved: all adult HF patients with Ejection Fraction (EF) <40% who are eligible for spironolactone (HF patients with New York Heart Association [NYHA] class II-IV. Should have creatinine <2.5mg/dL in men or <2.0 mg/dL in women, or estimated glomerular filtration rate [eGFR] >30 mL/min/1.73m2 and potassium <5.0 mEq/L). Exclusion criteria were: pediatrics, end-stage renal disease (since it is contraindicated to use spironolactone when creatinine clearance <30 ml/min), primary aldosteronism (since spironolactone can be used for different indication), and allergy to spironolactone. Outcomes variables include serum creatinine, potassium, eGFR, blood pressure (BP), whether the patient is on spironolactone, its dose, and if it is discontinued or not. The Institutional Review Board (IRB) approved our study (RC number: 17/101/R). After IRB approval, we queried data from a randomly selected sample of around 5000 HF patients from January 2016 to January 2017 by using the electronic system BestCare to generate a list of patients to be screened to meet our inclusion and exclusion criteria. The final study population was 368 patients.

Statistical analysis

Descriptive statistics were used to describe the study population. Data were analyzed using the appropriate descriptive statistics for the primary and secondary endpoints. All analyses were done using Statistical Product and Service Solutions (SPSS) Statistics for Windows, version 23 (IBM Corp., Armonk, NY). Socio‐demographic and clinical characteristics of participants are presented according to spironolactone use (yes vs no). To compare continuous data (e.g. serum creatinine) for the two groups, an independent t-test was used. The Chi-Square test was used to evaluate nominal data (e.g. patient comorbidities). A p-value below 0.05 is considered statistically significant in this study.

Multivariate regression analyses were performed to identify variables associated with spironolactone utilization. All variables were investigated including (age, gender, ejection fraction, NYHA classifications, body mass index, heart rate, systolic/diastolic blood pressure, history (Hx) of congestive HF or ischemic, diabetes mellitus, hypertension, dyslipidemia, smoking, asthma, atrial fibrillation, venous thromboembolism, stroke, chronic obstructive pulmonary disease, anemia, angina, unstable angina, myocardial infarction, family history (FHx) of coronary artery disease (CAD), medications: ACEI, ARBs, BB, aspirin, clopidogrel, furosemide, hydrochlorothiazide, indapamide, statin, hydralazine, isosorbide dinitrate, warfarin, and digoxin; and labs: sodium, potassium, creatinine, eGFR, and B-type natriuretic peptide (BNP). After that, due to the small sample size, we did backward elimination to omit factors that were not significantly associated with spironolactone use to end up with the following factors (age, EF, systolic blood pressure [SBP], diabetes, anemia, FHx of CAD, ACEI, furosemide, indapamide, statin, hydralazine, warfarin, digoxin, potassium, creatinine, and eGFR).

## Results

Between January 2016 and January 2017, 368 HFrEF patients were included from the screening of around 5000 heart failure patients. The use of spironolactone was 173 (47.01%) patients while 195 (52.99%) were not on it.

Baseline characteristics of the study population are shown in Table 1. The mean age of patients on spironolactone was 61±14 and the mean age of patients not on spironolactone was 66.6±15.6. Two hundred and seventy-seven patients in the study population were male. As for clinical variables, those who had EF <30 had more likelihood of going on spironolactone than patients with EF 30-40 where more were not on it. Also, patients who were on spironolactone had more control on SBP 121±19. In comorbidities, 265 patients in the study population were diabetic. Additionally, patients with an FHx of CAD had more prescriptions for spironolactone (12 patients) than those with a negative FHx (three patients). Moreover, medications that were used more concomitantly with spironolactone were ACEI, furosemide, warfarin, and digoxin while indapamide, statins, and hydralazine were used less concomitantly. As for laboratory findings, the mean serum potassium for patients on spironolactone was 4.3 mEq/L; the creatinine and eGFR for patients on spironolactone 82 umol/L (0.9 mg/dl) and 88 mL/min/1.73m2 eGFR while those not on spironolactone had higher creatinine 93 umol/L (1 mg/dl) and 80 mL/min/1.73m2 eGFR; the BNP was higher in patients on spironolactone 153 pmol/L and 109 pmol/L in patients not on spironolactone.

**Table 1:** Baseline Characteristics SBP: systolic blood pressure, SD: standard deviation, CAD: coronary artery disease, ACEI: angiotensin-converting enzyme inhibitor, eGFR: estimated glomerular filtration rate, BNP: B-type natriuretic peptide

Table 1						
Baseline Characteristics						
Variables		On Spironolactone n=173		Not On Spironolactone n=195		
Demographics						P-Value
Age (± SD), years		61.0 (14.2)		66.6 (15.7)		<0.01
Gender						0.20
Male n (%)		125 (33.9)		152 (41.3)		
Clinical Variables						
Ejection Fraction Before Spironolactone						<0.01
<30 n (%)		85 (23.1)		58 (15.8)		
30 – 40 n (%)		88 (23.9)		137 (37.2)		
SBP (± SD), mmHg		120.9 (19.2)		125.7 (18.1)		0.01
Co-Morbidities						
Diabetes						<0.01
Yes n (%)		113 (30.7)		152 (41.3)		
Anemia						0.04
Yes n (%)		4 (1.09)		13 (3.50)		
Family History of CAD						<0.01
Yes n (%)		12 (3.26)		3 (0.82)		
Medications						
ACEI						0.03
Yes n (%)		112 (30.4)		105 (28.5)		
Furosemide						<0.01
Yes n (%)		130 (35.3)		96 (26.1)		
Indapamide						0.04
Yes n (%)		1 (0.27)		7 (1.90)		
Statin						0.01
Yes n (%)		151 (41.0)		184 (50.0)		
Hydralazine						<0.01
Yes n (%)		5 (1.36)		19 (5.16)		
Warfarin						0.01
Yes n (%)		22 (5.98)		11 (2.99)		
Digoxin						0.06
Yes n (%)		13 (3.53)		6 (1.63)		
Laboratory Findings						
Baseline Potassium – mEq/L		4.3 (0.41)		4.4 (0.42)		0.07
Baseline Creatinine – umol/L		81.2 (23.4)		92.7 (38.3)		<0.01
Baseline eGFR - mL/min/1.73m^2^		88.0 (23.7)		79.6 (27.3)		<0.01
Baseline BNP pmol/L		153 (218)		109 (173)		0.06

**Figure 1:**
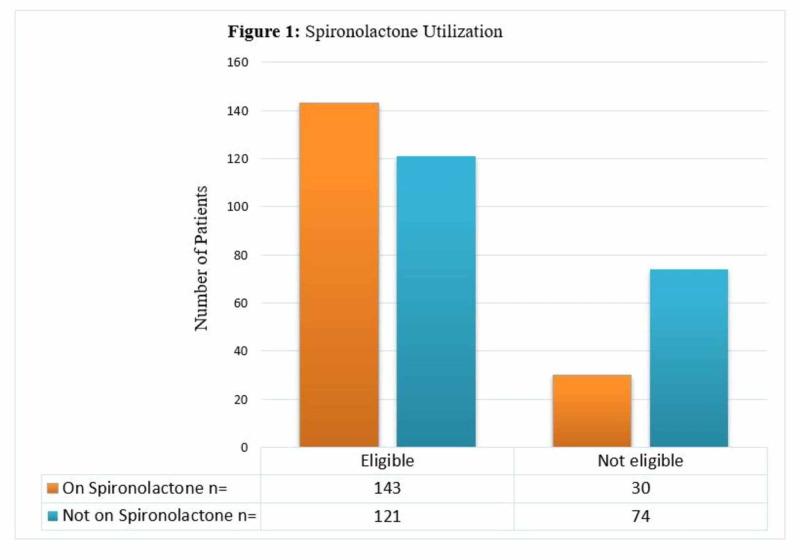
Spironolactone Utilization

As for the utilization of spironolactone (Figure 1), among 195 patients who were not on spironolactone, 121 patients had eligibility to use it; however, they did not receive it. One hundred seventy-three patients were on spironolactone, of whom 30 patients received the drug although they did not meet the eligibility criteria.

Moreover, in Table 2, using multivariate logistic regression to find independent factors associated with the underutilization of spironolactone, we found the following factors to be significant: EF before spironolactone (odds ratio [OR]=2.56 [95% CI: 1.40-4.69, P=<0.01]), serum creatinine (OR=1.01 [95% CI: 1.00-1.02, P=0.02]), ACEI (OR=7.46 [95% CI: 2.45-22.7, P=<0.01]), ARBs (OR=6.54 [95% CI: 2.01-21.3, P=<0.01]), furosemide (OR=3.54 [95% CI: 1.90-6.62, P=<0.01]), statins (OR=0.18 [95% CI: 0.05-0.61, P=<0.01]), stroke (OR=2.86 [95% CI: 1.00-8.17, P=0.05]).

**Table 2:** Independent Association Using Logistic Regression ACEI: angiotensin-converting enzyme inhibitor, ARBS: angiotensin-II receptor antagonists

Variables	Odds Ratio (95% CI)	P-Value
Ejection Fraction Before Spironolactone	2.56 (1.40-4.69)	<0.01
Stroke	2.86 (1.00-8.17)	0.05
ACEI	7.46 (2.45-22.7)	<0.01
ARBS	6.54 (2.01-21.3)	<0.01
Furosemide	3.54 (1.90-6.62)	<0.01
Statin	0.18 (0.05-0.61)	<0.01
Serum Creatinine	1.01 (1.00-1.02)	0.02

## Discussion

To the best of our knowledge, this is the first study to assess spironolactone utilization in HFrEF patients in Saudi Arabia. In this cross-sectional retrospective study, spironolactone was underutilized for indicated HF patients. The factors associated with the underutilization of spironolactone were EF, serum creatinine (although it was within an acceptable range to use spironolactone), ACEI, ARBs, furosemide, statins, and stroke.

Spironolactone underutilization in this study is consistent with previous studies. In a registry that was done between 2005-2007, they found that patients eligible to use AA represent only 32.5% of the study population [18]. In the Improve the Use of Evidence-Based Heart Failure Therapies in the Outpatient Setting (IMPROVE HF) registry, the use of AA was to 36% of 2505 eligible patients [19]. Similarly, in the EuroHeart Failure Survey II (EHFS II), 47.5% of patients used AA upon discharge after admission with HF [22]. Furthermore, in findings from BIOSTAT-CHF, a European multicenter, prospective observational study, only 56% of eligible patients received AA at baseline which improved to 63% after following up with patients as part of an HF treatment optimization program [23]. Also, in the Medicare-linked Organized Program To Initiate Lifesaving Treatment In Hospitalized Patients With Heart Failure (OPTIMIZE-HF) registry, 6986 from 8206 patients had eligibility to use spironolactone based on serum creatinine; however, they were not on it [24]. Additionally, in the Swedish Heart Failure Registry (SwedeHF) 11,215 patients, only 4443 (40%) of eligible patients received an AA, and to find factors associated with underutilization of AA where creatinine was found to be significantly associated with low-use (although within an acceptable range) while serum potassium was not significant [25]. In our study, the rate of using spironolactone in eligible HF patients was 39%.

Several factors have been associated with spironolactone underutilization in this study, including EF before spironolactone, since patients with HFrEF have a stronger recommendation to use AA to improve survival; another factor is serum creatinine, as the hyperkalemia risk increases with worsening renal function; ACEI and ARBs both limited the use of spironolactone due to the effect of hyperkalemia they can cause; furosemide, which could be due to the risk of over diuresis/dehydration, statin, and stroke; where most patients are on ACEI/ARBs leading to an increased risk of hyperkalemia [26]. In addition to the factors limiting spironolactone use, we plan to do another study to find barriers limiting spironolactone utilization from the prescribers’ perspective, assessing the knowledge, perception, and comprehension of the ACCF/AHA and ESC guidelines in regard to AA use among HF patients.

This study has some limitations that need to be identified, for example the small sample size of the study population, in addition to the single-center setting, although the institution (KAMC) is considered one of the leading hospitals in Saudi Arabia in cardiovascular medicine. Another limitation is the retrospective nature of the study, therefore the causality effect cannot be applied. Moreover, when using regression, a stepwise selection was utilized due to the lack of sufficient sample size, which might have an effect on the accuracy of the results.

## Conclusions

The use of spironolactone for HF patients is underutilized. EF before spironolactone, serum creatinine, ACEI, ARBs, furosemide, statin, and stroke significantly affected spironolactone utilization. The findings of this study indicate that there is a gap in utilizing spironolactone in Saudi Arabia, which implicates the necessity to find barriers limiting its use and make protocols to assure the compliance with the guidelines. Further studies are warranted to identify other factors and potential barriers from the prescribers' perspectives affecting the utilization of spironolactone in HF patients.
